# BacTag - a pipeline for fast and accurate gene and allele typing in bacterial sequencing data based on database preprocessing

**DOI:** 10.1186/s12864-019-5723-0

**Published:** 2019-05-06

**Authors:** Lusine Khachatryan, Margriet E. M. Kraakman, Alexandra T. Bernards, Jeroen F. J. Laros

**Affiliations:** 1 Department of Human Genetics, Leiden University Medical Center, Leiden, The Netherlands; 2 Department of Medical Microbiology, Leiden University Medical Center, Leiden, The Netherlands; 3 Clinical Genetics, Leiden University Medical Center, Leiden, The Netherlands; 4 GenomeScan, Leiden, The Netherlands

**Keywords:** Next-generation sequencing, Multi-locus sequence typing, Database preprocessing, Allele typing

## Abstract

**Background:**

Bacteria carry a wide array of genes, some of which have multiple alleles. These different alleles are often responsible for distinct types of virulence and can determine the classification at the subspecies levels (e.g., housekeeping genes for Multi Locus Sequence Typing, MLST). Therefore, it is important to rapidly detect not only the gene of interest, but also the relevant allele. Current sequencing-based methods are limited to mapping reads to each of the known allele reference, which is a time-consuming procedure.

**Results:**

To address this limitation, we developed BacTag - a pipeline that rapidly and accurately detects which genes are present in a sequencing dataset and reports the allele of each of the identified genes. We exploit the fact that different alleles of the same gene have a high similarity. Instead of mapping the reads to each of the allele reference sequences, we preprocess the database prior to the analysis, which makes the subsequent gene and allele identification efficient. During the preprocessing, we determine a representative reference sequence for each gene and store the differences between all alleles and this chosen reference. Throughout the analysis we estimate whether the gene is present in the sequencing data by mapping the reads to this reference sequence; if the gene is found, we compare the variants to those in the preprocessed database. This allows to detect which specific allele is present in the sequencing data. Our pipeline was successfully tested on artificial WGS *E. coli, S. pseudintermedius, P. gingivalis, M. bovis, Borrelia spp. and Streptomyces spp.* data and real WGS *E. coli* and *K. pneumoniae* data in order to report alleles of MLST house-keeping genes.

**Conclusions:**

We developed a new pipeline for fast and accurate gene and allele recognition based on database preprocessing and parallel computing and performed better or comparable to the current popular tools. We believe that our approach can be useful for a wide range of projects, including bacterial subspecies classification, clinical diagnostics of bacterial infections, and epidemiological studies.

**Electronic supplementary material:**

The online version of this article (10.1186/s12864-019-5723-0) contains supplementary material, which is available to authorized users.

## Background

In order to understand and predict the pathogenic impact and the outbreak potential of a bacterial infection, knowing the species responsible for this infection is not sufficient. Bacterial virulence is often controlled on the sub-species level by the set of specific genes or sometimes even alleles, leading to the necessity of diverse treatment strategies for infections induced by the same bacterial species [1–5]. For example, antibiotic resistance is one of the most well-known examples where slight variations in a gene can lead to a vast collection of antibiotics resistance profiles within one taxonomic group [6, 7]. Furthermore, different alleles of the same gene can be responsible for distinct adhesion and invasion strategies, reactions to the immune response of the infected organism and toxin production [8, 9]. Besides its relevance for understanding virulence, finding the alleles of specific genes also contributes to a more accurate bacterial classification. One of the most popular methods for subspecies bacterial typing, MultiLocus Sequence Typing (MLST), is based on determination of the alleles of multiple housekeeping genes [10, 11]. Knowing the allele combination allows to identify so called Sequencing Type (ST) of the organism, which is often associated with the important pathogen’s attributes such as infection potential [12–14] or the ability to cause disease in human by transmitting from their animal reservoirs [15–17]. MLST typing is crucial for the epidemiological studies as it provides fast and accurate identification of geographical dispersal of pathogens and even reveals the migration patterns of the host organism [18, 19].

Despite the importance of the gene and allele typing in the bacterial genomes, there is no “gold standard” method to perform it. For a long time, the presence of particular virulent genes was detected using phenotypic markers such as serotyping [20]. Unfortunately, the set of genetic features that can be revealed using only the phenotype is very limited. Among other restrictions of this group of methods are the inability to grow certain fastidious pathogens in laboratory conditions as well as the extensive delay in cultivation and identification for slowly growing pathogens [21–25]. In particular cases, the gene and allele identification problem can be solved by using PCR or microarrays with gene- and allele specific primers or probes [26–28]. These types of methods are much faster and more reliable in comparison to the phenotype-based approaches. However, for the vast majority of genes it is impossible to generate primers or probes that would perform the allele discrimination due to the high similarity among sequences of alleles. Thus, PCR based typing often needs additional analysis, for example, a restriction fragment length polymorphism typing [29, 30] which elaborates the analysis process. PCR-based gene and allele typing most of the time has to be “tailor-made” for the particular group of organisms and the gene of interest. The rapid growth of newly discovered bacteria together with the high mutation rate of some genes causes the necessity of constant changes in the existing PCR-protocols.

With the improvement of high throughput sequencing techniques and the development of associated bioinformatics software, it became possible to identify the allele variations directly from Whole Shotgun Genome Sequencing (WGS) data by comparing sequencing reads to the reference sequences of the known alleles of the gene of interest in the curated database. Currently, most of the curated and publicly available databases suitable for the gene typing are designed for subspecies classification using the MLST principle. These databases contain variable alleles of housekeeping genes and MLST schemas, associated with those housekeeping genes, for more than 60 bacterial species [31]. There are several tools that perform MLST by aligning assembled WGS data to each sequence in the linked database and reporting the alleles of housekeeping genes with the highest similarity to the provided data [32, 33]. The most recent tools for automated MLST performs the analysis on raw WGS data, as the assembly step is included in its pipeline [34, 35]. Finally, stringMLST software [36] performs allele identification by comparing the k-mer profiles of raw sequencing data to the *k*-mer profiles of sequences in the MLST database. This strategy allows to speed up the analysis process drastically, yet the accuracy of the method is lower in comparison with alignment-based ones [37].

Though the WGS-based methods for gene and allele typing potentially requires less effort than any laboratory technique, it has some disadvantages and room for improvement. First of all, the time-consuming separate alignment of WGS data to each sequence in the database can be substituted with a faster algorithm. Furthermore, most of the existing bioinformatics tools for MLST do not provide an option to optimize the analysis settings, which means that the user cannot control, for example, parameters of reads mapping. Finally, it is also not possible to perform the analysis using a database or MLST schema that is not associated with the tool.

In this paper we present BacTag (**Bac**terial **T**yping of **a**lleles and **g**enes) - a new pipeline, designed to rapidly and accurately detect genes and alleles in sequencing data. Due to the database preprocessing prior to the analysis, BacTag providing a solid and more detailed basis for downstream in comparison with similar tools while retaining the same accuracy. Additionally, our method performs gene and allele detection slightly faster than its current analogs. Our pipeline was successfully tested on both artificial (*E. coli, S. pseudintermedius, P. gingivalis, M. bovis, Borrelia spp. and Streptomyces spp.*) data and real (*E. coli, K. pneumoniae*) clinical WGS samples, by preprocessing the corresponding MLST databases and by performing the subsequent typing. This method is publicly available at https://git.lumc.nl/l.khachatryan/BacTag.

## Methods

### Pipeline implementation

The user interface is implemented in Bash, the processing modules are written in GNU Make. Bash allows for user interaction and files maintenance, while GNU Make makes the pipeline suitable for parallel high-performance computing. The pipeline consists of two parts: database preprocessing and sequencing data analysis. Both parts contain modules that include published tools and the scripts from our Python library. The pairwise sequence alignment is performed by the *aln* command from fastools [38]. Artificial paired end Illumina FASTQ formatted reads are created by the *make_fastq local* command of sim-reads [39]. Reads are mapped to a reference sequence with BWA *mem* [40]. Alignment sorting and indexing are performed by SAMtools [41]. Potential PCR duplicates are removed using SAMtools *rmdup* command. The SAMtools *mpileup* utility is used to summarize the coverage of mapped reads on a reference sequence at single base pair resolution. Variant calling is performed by the *call* command of BCFtools [42]. To verify whether the called variants for each allele really correspond to the allele sequence, the *vcf-consensus* command of VCFtools [43] is used. Comparison of two VCF files boils down to reporting the number of variants sites that are not equal for both files. Programming languages and software versions used for pipeline construction can be found in Additional file 1: Table S1. The user may specify parameters for artificial reads generation (by default read length, insert size and coverage are equal 50 nucleotides, 100 nucleotides and 40 respectively), the BWA *mem* and SAMtools *mpileup* utilities for both database preprocessing and sequencing data analysis parts separately. It is also possible to set the ploidy (by default this is one) of the sequencing data, which will be considered during the variants calling in the analysis part of the pipeline.

### Database preprocessing

**Fig. 1:**
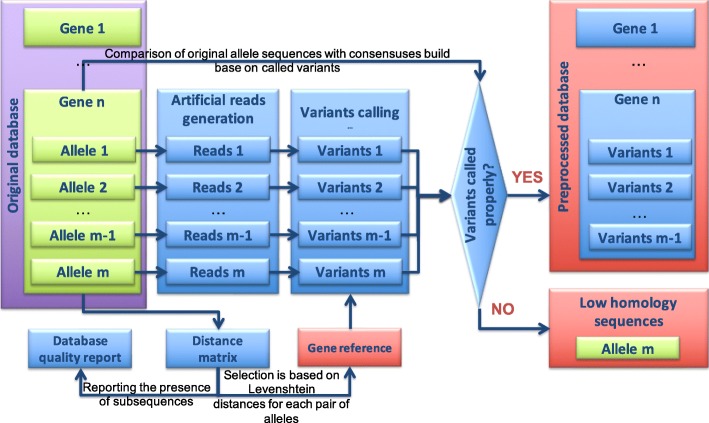
Schematic representation of the database preprocessing. All of the processes are illustrated for one gene. Calculations for several genes are done independently in parallel

The database preprocessing workflow is shown in Fig. 1. We designed the pipeline such that all independent processes are performed in parallel, which reduces the calculation time.

The user provides the database that consists of alleles grouped by genes of interest. Optionally, the user can provide the 5′- and 3′-flanking regions for each gene, otherwise, every allele will be flanked on both sides with a fifty-nucleotide long poly-N sequence. That is done in order to prevent the coverage drop at the end of sequence during the sequencing data mapping. In the first step of the preprocessing stage, the sequences of all alleles belonging to the same gene are aligned in a pairwise manner, yielding the Levenshtein [44] distance for each pair of alleles. These distances are used to select the allele with the smallest average distance to all other sequences as the gene reference. In the same step the quality of the provided database is checked: it is reported when the same sequence is provided for multiple alleles or when one allele sequence is a subsequence of another. Once the quality report is created, the user can fix the original database when needed. In the next step, artificial Illumina paired end reads are created based on the sequence of each allele. Reads are mapped to the selected gene reference, the alignment map file is sorted and indexed, after which the coverage of mapped reads on the reference sequence at a single base pair resolution is summarized and stored in a BCF file, which is used for variants calling. Variants are stored in a VCF file and further subjected to a quality check to verify whether they really correspond to the allele sequence. If variants defining allele’s sequence were not properly called, allele is reported and assigned to the so-called low similarity group of sequences. The low similarity group contains sequences for which the variants were not called correctly during the database preprocessing when using the centroid reference. I.e., for these alleles, the centroid is not an appropriate reference and therefore these sequences should be considered to be references themselves. In the final step the references of all genes are concatenated into one FASTA file, which further serves as the database reference.

### Sequencing data analysis

**Fig. 2:**
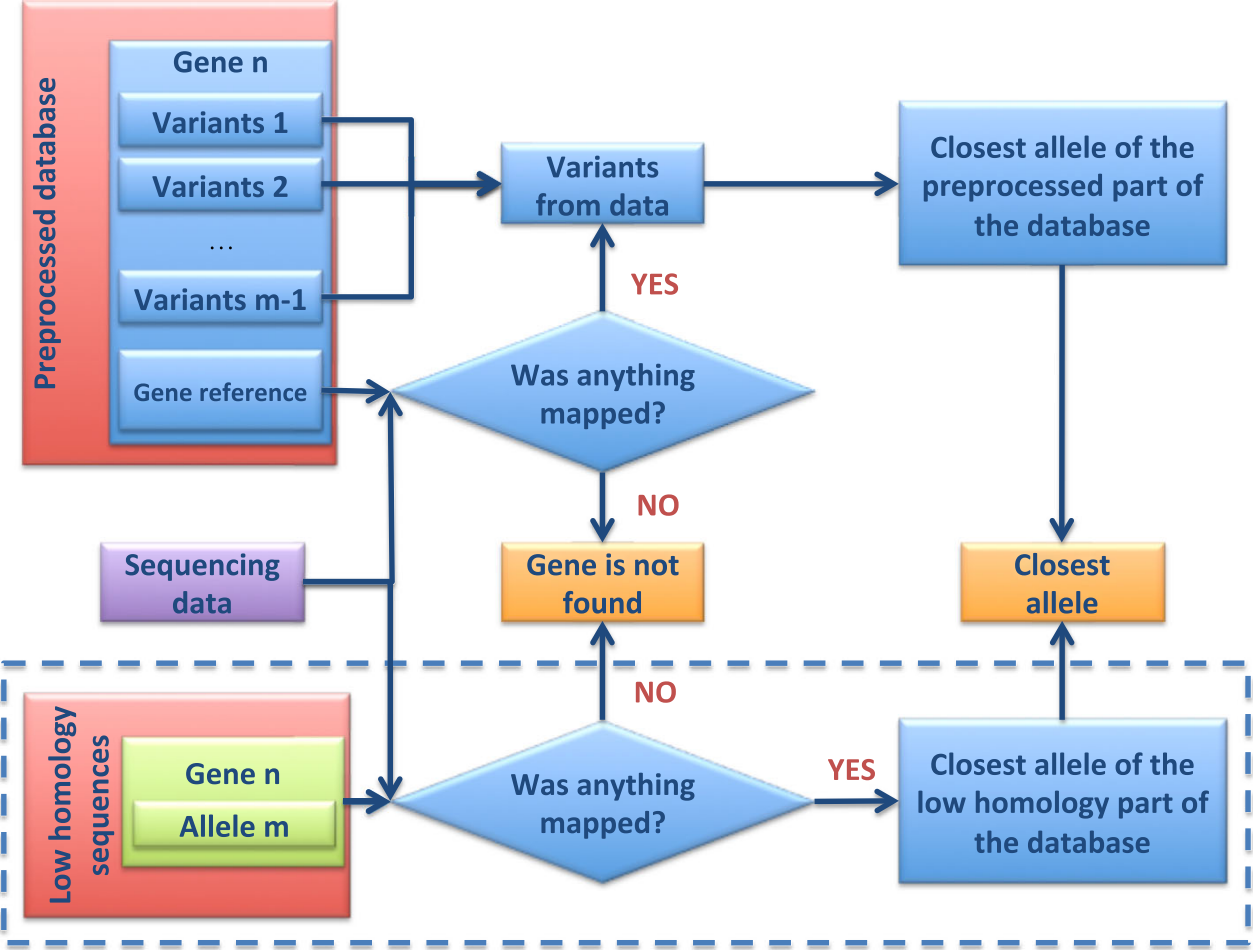
Schematic representation of the analysis part of BacTag pipeline. All of the processes are illustrated for one gene. Calculations for multiple genes are done independently in parallel. The analysis of the low similarity group of sequences is highlighted by the dashed box and can be manually turned off by the user for the time efficiency

The data analysis workflow can be found in Fig. 2. To initiate the analysis, the user provides two paired FASTQ files. After analysis initialization an output directory is created, which will serve to store the results of the analysis. The user can choose the name of the output directory, otherwise it will have the same name as the basename of the provided FASTQ files. The sequencing data analysis part of the pipeline is comprised of two steps: the main analysis and the analysis of low similarity group of sequences. If no sequences were assigned to the low similarity group during the database preprocessing, only the first step will be performed. The user can manually turn off the second step for time efficiency.

#### The main analysis

This part of the pipeline applies to the alleles that were not placed in the low similarity group of sequences during the database preprocessing. Analyzed reads are mapped to the database reference, obtained after database preprocessing by concatenating all the gene reference sequences. The alignment map file is indexed and sorted and substituted to the removal of potential PCR duplicates. If there are no reads mapped to the gene reference, the gene is reported as not found in the analyzed dataset. Otherwise, mapped reads are used to estimate the horizontal coverage of a gene reference at base pair resolution. The obtained BCF coverage summary is used for variant calling, the result of which is stored in VCF format. Variants are compared with variants collected for each gene allele during the preprocessing phase. Once the comparisons are done, the allele with the least difference from the sequencing data will be reported. Multiple variants at the same position are also reported, as this might indicate sequencing or mapping problems as well as the presence of more than one allele of the same gene in the sequencing data. Reports for all genes are concatenated to a single result file, which is placed in the output directory.

#### Low similarity group of sequences analysis

This part of the pipeline works with alleles that were assigned to the low similarity group of sequences during the database preprocessing. Sequencing reads are subjected to variant calling using each of the alleles from the low similarity group as a reference (the same routine with the same parameters as for the main analysis step). If for the particular gene one of the alleles from the low similarity group has fewer differences with the sequencing data in comparison to the allele reported during the main analysis, the allele from the low similarity group will be reported as present in the sequencing data.

### Pipeline testing

All the computational benchmarking was done on chimerashark Blade Server of SHARK computer cluster [45] with the maximum of 24 CPUs used at the same time.

#### Database

##### Genes and alleles

The database preprocessing part of the pipeline was tested using seven curated databases: *E. coli* Achtman MLST ([46], downloaded January 2018), *K. pneumoniae* Pasteur MLST ([47], downloaded October 2018), *S. pseudintermedius* MLST ([48], downloaded February 2019), *P. gingivalis* MLST ([49], downloaded February 2019), *M. bovis* MLST ([50], downloaded February 2019), *Borrelia spp.* MLST ([51], downloaded February 2019) *and Streptomyces spp.* MLST ([52], downloaded February 2019). Each database contains sequences of variable regions of housekeeping genes: five for the *Streptomyces spp.* MLST, eight for the *Borrelia spp.* MLST and seven for all the remaining schems (see Table 1).

**Table 1:** Preprocessed MLST databases

MLST database	Genes including number of alleles per gene	Number of alleles (per gene) in the low similarity group	Strain and reference sequence we used for flanking region construction
*E. coli*	*adk (*623), *fumC (*933), *gyrB (*606), *Icd (823), mdh (614), purA (563), recA (512)*	*fumC (11*), *gyrB (3*), *mdh (8)*	UMN026, NC_011751.1
*K. pneumoniae*	*gapA (184), infB (141), mdh (245), pgi (221), phoE (365), rpoB (189), tonB (472)*	*gapA (6), mdh (3), tonB (29)*	Kp52.145, FO834906.1
*S. pseudintermedius*	*ack (46), cpn60 (96), fdh (26), pta (70), purA (77), sar (38), tuf (24)*	–	ED99, NC_017568.1
*M. bovis*	*adh1 (15), gltX (17), gpsA (14), gyrB (25), pta2 (23), tdk (15), tkt (26)*	–	PG45, NC_014760.1.
*P. gingivalis*	*ftsQ (40), gpdxJ (37), hagB (37), mcmA (30), pepO (37) pga (27), recA (14)*	–	ATCC 33277, NC_010729.1
*Borrelia spp.*	*clpA (296), clpX (258), nifS (230), pepX (261), pyrG (269), recG (285), rplB (250), uvrA (261)*	*clpA (58), clpX (51), nifS (54), pepX (57), pyrG (51), recG (55), rplB (54), uvrA (45)*	*B. hermsii* DAH, NC_010673.1
*Streptomyces spp.*	*atpD (183), gyrB (179), recA (184), rpoB (183), trpB (200)*	*atpD (72), gyrB (147), recA (2), rpoB (6), trpB (69)*	*S. coelicolor* A3(2), NC_003888.3

MLST schemas were selected for organisms from six different bacterial phyla. These organisms have a GC-content ranging between 29 and 73%. For the database preprocessing the following parameters for BWA *mem* and SAMtools *mpileup* tools were selected. Since the database consists of sequences of highly variable regions of housekeeping genes, the alignment mismatch penalty was set to 2 (4 by default) in order to provide the proper alignment for the regions where variants occur in close proximity. The minimum seed length was changed to 15 (19 by default) due to the short length of sequences in the selected database. Penalty for 5′- and 3′-end clipping was set to 100 (5 by default), forcing alignment to detect the variants located at the ends of the variable region. Single end mapped reads (anomalous read pairs, −A) were counted in order to detect variants located at the ends of the variable region. BAQ computation was disabled, as it is oversensitive to regions densely populated with variants. Bases with baseQ/BAQ lower than 13 were not skipped, since the database preprocessing is based on high quality artificial sequencing reads.

##### Flanking regions

The sequences of polymerase chain reaction (PCR) primers commonly applied to amplify each of the housekeeping genes [53–57] for the selected MLST schemas were used to construct the flanking regions for this study. Each flanking region includes the primer sequence as well as the genomic sequence between the primer and the variable region of interest. The genomic sequence is extracted from the genome of one of the target strains for the corresponding MLST schema (see Table 1). In case low-sensitivity PCR primers are used (e.g., for *Borrelia spp.* MLST) or if no PCR primer sequences are available (e.g., for *Streptomyces spp.* MLST)*,* fifty nucleotides before and after the variable regions were used as flanks. Flanking regions have the same orientation as the allele sequences in the database (see Additional file 2: Tables S2-S8).

#### Artificial test data

The sequencing data analysis part of the pipeline was validated by using artificial Illumina reads, based on the complete genomes of 30 different bacterial strains belonging to 13 different bacterial species (see Table 2), for which the alleles of housekeeping genes associated with the corresponding MLST schema were previously reported. Paired end FASTQ formatted reads of 100 bp were generated with an insert size of 100. For each genome, an average coverage of 80 was generated in this way.

**Table 2:** Testing the pipeline on artificial WGS data

Species and strain	GeneBank Accession number	Identified alleles
*E. coli* 042	FN554766.1	*adk-18, fumC-22, gyrB-20, Icd-23, mdh-5, purA-15, recA-4*
*E. coli* E2348/69	FM180568.1	*adk-15, fumC-15, gyrB-11, Icd-15, mdh-18, purA-11, recA-11*
*E. coli* E24377A	CP000800.1	*adk-6, fumC-213, gyrB-33, Icd-1, mdh-24, purA-8, recA-7*
*E. coli* IHE3034	NC_017628.1	*adk-37, fumC-38, gyrB-19, Icd-37, mdh-17, purA-11, recA-26*
*E. coli* IMT5155	CP005930.1	*adk-55, fumC-38, gyrB-19, Icd-37, mdh-17, purA-11, recA-26*
*E. coli* RS218	NZ_CP007149.1	*adk-37, fumC-38, gyrB-19, Icd-37, mdh-17, purA-11, recA-26*
*E. coli* UMN026	NC_011751.1	*adk-21, fumC-35, gyrB-115, Icd-6, mdh-5, purA-5, recA-4*
*S. pseudintermedius* NA45	NZ_CP016072.1	*ack-2, cpn60–10, fdh-2, pta-1, purA-5, sar-1, tuf-2*
*S. pseudintermedius* ED99	NC_017568.1	*ack-3, cpn60–9, fdh-2, pta-1, purA-1, sar-1, tuf-1*
*S. pseudintermedius* HKU10–03	NC_014925.1	*ack-2, cpn60–55, fdh-3, pta-42, purA-14, sar-2, tuf-1*
*M. bovis* Ningxia-1	NZ_CP023663.1	*adh1–4, gltX-3, gpsA-2, gyr-3, pta2–17, tdk-3, tkt-4*
*M. bovis* HB0801	NC_018077.1	*adh1–4, gltX-3, gpsA-2, gyr-3, pta2–5, tdk-3, tkt-4*
*M. bovis* NM2012	NZ_CP011348.1	*adh1–4, gltX-3, gpsA-2, gyr-3, pta2–5, tdk-3, tkt-4*
*M. bovis* CQ-W70	NC_015725.1	*adh1–4, gltX-5, gpsA-2, gyr-3, pta2–5, tdk-3, tkt-4*
*M. bovis* PG45	NC_014760.1	*adh1–3, gltX-2, gpsA-4, gyr-2, pta2–1, tdk-3, tkt-2*
*M. bovis* 08 M	NZ_CP019639.1	*adh1–4, gltX-3, gpsA-2, gyr-3, pta2–5, tdk-3, tkt-4*
*P. gingivalis* ATCC 33277	NC_010729.1	*ftsQ-5, gpdxJ-9, hagB-1, mcmA-1, pepO-1, pga-5, recA-5*
*P. gingivalis* AJW4	NZ_CP011996.1	*ftsQ-21, gpdxJ-23, hagB-1, mcmA-3, pepO-20, pga-3, recA-7*
*P. gingivalis* A7A1–28	CP013131.1	*ftsQ-1, gpdxJ-12, hagB-1, mcmA-1, pepO-1, pga-1, recA-1*
*Borrelia hermsii* DAH	NC_010673.1	*clpA-68, clpX-165, nifS-149, pepX-171, pyrG-179, recG-188, rplB-157, uvrA-175*
*Borrelia turicatae* 91E135	NC_008710.1	*clpA-71, clpX-166, nifS-150, pepX-172, pyrG-180, recG-189, rplB-158, uvrA-176*
*Borrelia anserina* BA2	CP005829	*clpA-212, clpX-179, nifS-161, pepX-186, pyrG-196, recG-204, rplB-170, uvrA-188*
*Borrelia recurrentis* A1	NC_011244	*clpA-213, clpX-164, nifS-162, pepX-187, pyrG-197, recG-205, rplB-156, uvrA-189*
*Borrelia parkeri* SLO	CP005851	*clpA-214, clpX-180, nifS-163, pepX-188, pyrG-198, recG-206, rplB-171, uvrA-190*
*Borrelia coriaceae* Co53	CP005745	*clpA-215, clpX-181, nifS-164, pepX-189, pyrG-199, recG-207, rplB-172, uvrA-191*
*Borrelia crocidurae* Achema	CP003426	*clpA-216, clpX-164, nifS-165, pepX-190, pyrG-200, recG-208, rplB-173, uvrA-192*
*Streptomyces coelicolor A3(2)*	NC_003888.3	*atpD-127, gyrB-124, recA-131, rpoB-126, trpB-142*
*Streptomyces fulvissimus* DSM 40593	CP005080.1	*atpD-133, gyrB-130, recA-13, rpoB-36, trpB-147*
*Streptomyces griseus* NBRC 13350	NC_010572.1	*atpD-6, gyrB-8, recA-8, rpoB-8, trpB-8*
*Streptomyces albidoflavus* J1074	NC_020990.1	*atpD-36, gyrB-5, recA-5, rpoB-36, trpB-39*

#### Real test data

The analysis part of the pipeline was tested on 185 paired end Illumina WGS samples belonging to nine different previously reported sequencing types (STs) of *E. coli* (see Additional file 3: Table S9) and 98 paired end Illumina WGS samples belonging to 43 different previously reported STs of *K. pneumoniae* (see Additional file 3: Table S10). Sequencing reads were downloaded from Sequence Read Archive (SRA, [58]). Prior to the analysis, the data quality check and correction (when necessary) was done for each sample using Flexiprep QC pipeline [59].

#### Parameters used for sequencing data analysis

The analysis of both artificial and real samples was done with the same parameters of BWA *mem* as during the database preprocessing. SAMtools *mpileup* parameters were as follow: anomalous read pairs were counted; extended BAQs were calculated for higher sensitivity but lower specificity.

## Results

### Building the preprocessed MLST databases

We used BacTag to preprocess seven publicly available MLST databases. During this process we did not detect any duplications or partial sequences for any of the preprocessed databases.

When preprocessing *E. coli* Achtman seven genes MLST database, 22 sequences (less than 0.5% of the total number of analyzed sequences) belonging to three different genes were assigned to the low similarity group of sequences (see Table 1). The run time of the *E. coli* database preprocessing was approximately 2 h. The peak memory usage was 150 Mb. During the preprocessing of the *K. pneumoniae* database associated with the Pasteur seven genes MLST schema, 38 sequences (2.1% of the total number of analyzed sequences) belonging to three different genes were assigned to the low similarity group of sequences. Preprocessing of databases associated with MLST schemas for *S. pseudintermedius, M. bovis* and *P. gingivalis* reported no sequences placed in the low similarity group of sequences. For the databases associated with the MLST schemas for *Borrelia spp.* and *Streptomyces spp.* 425 sequences (19.2% of the total number of analyzed sequences) and 296 sequences (31.8% of the total number of analyzed sequences) were placed in the low similarity group respectively. This large number of low similarity sequences indicates that the alleles in the analyzed MLST databases are quite heterogeneous, which can be expected, considering that both aforementioned MLST schemas are genus-specific, not species-specific like other five analyzed databases.

**Fig. 3:**
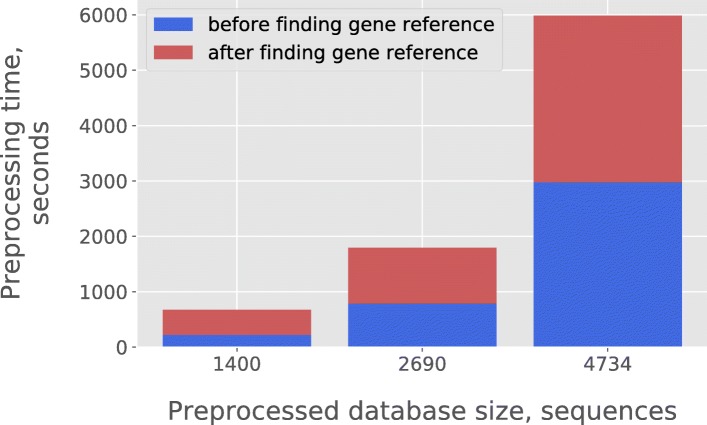
The dependence of database preprocessing time from the amount of sequences in the database

Since distance matrix is computed during the preprocessing, the expected CPU time will scale quadratically with the size of the database. We indeed found this behavior as shown in Fig. 3.

### Testing BacTag on artificial data

We used the preprocessed MLST databases to reveal the presence of the corresponding housekeeping genes and to predict the allele for each of these genes in artificial sequencing data based on complete genomes of 30 different bacterial strains belonging to 15 different species. All housekeeping genes associated with the corresponding MLST schema were identified in each sample. The alleles found by the pipeline matched with the previously reported ones for each but one of the analyzed genomes (Table 2). The genome of *P.gingivalis* AJW4 (GenBank accession number NZ_CP011996.1) was previously reported [60] to have the allelic variants *ftsQ-16, gpdxJ-9, hagB-22, mcmA-17, pepO-22, pga-15 and recA-1*. However, BacTag analysis revealed the following set of alleles: *ftsQ-21, gpdxJ-23, hagB-1, mcmA-3, pepO-20, pga-3 and recA-7.* Manual inspection confirmed that alleles reported by BacTag are correct in case of all aforementioned genes.

### Testing BacTag on real *E. coli* and *K. pneumoniae* data

We tested BacTag on 185 *E. coli* and 97 *K. pneumoniae* clinical publicly accessible WGS datasets, with each test yielding either one of nine *E. coli* or one of 44 *K. pneumoniae* sequencing types (STs). *E. coli* samples were analysed using the preprocessed *E. coli* Achtman seven genes MLST database, while *K. pneumoniae* samples were analysed using the preprocessed *K. pneumoniae* Pasteur seven genes MLST database. Each sample was shown to contain all expected seven housekeeping genes; alleles of those genes identified using our method corresponded to the expected ones for all but one sample (Table 3). This sample was checked additionally using web-based tools for the MLST [34, 35]. Results of this independent check were completely identical to the ones obtained by our pipeline and suggest that the sample belongs to *E. coli* ST95 instead of ST73. Furthermore, according to the original publication [61], MLST was never done for this and 21 other samples analyzed during the same study in order to confirm their sequencing type. Thus, we conclude that in Ref. [60] one of the samples was incorrectly assigned to *E.coli* ST73.

**Table 3:** Results of pipeline testing on real *E. coli* and *K. pneumoniae* data. Only samples with results different from expected are shown

SRA Run AC	Reported ST	Expected ST	Genes with multiple variants at the same position
ERR966604	95	73	–
SRR2767732	16	16	*Icd*
SRR2767734	21	21	*Icd, mdh*
SRR2970643	131	131	*fumC*
SRR2970737	131	131	*adk, fumC, gyrB, mdh, recA, purA*
SRR2970742	131	131	*fumC*
SRR2970753	131	131	*fumC*
SRR2970774	131	131	*fumC*
SRR2970775	131	131	*fumC*
SRR5973405	1164	1164	*phoE*
SRR5973308	1180	1180	*phoE*
SRR5973303	13	13	*phoE*
SRR5973253	133	133	*phoE*
SRR5973334	133	133	*phoE*
SRR5973324	1373	1373	*phoE*
SRR5973251	1426	1426	*gapA, phoE*
SRR5973269	147	147	*gapA*
SRR5973320	1876	1876	*phoE*
SRR5973351	188	188	*gapA*
SRR5973329	20	20	*phoE*
SRR5973408	2267	2276	*phoE*
SRR5973397	25	25	*phoE*
SRR5973248	258	258	*gapA*
SRR5973283	258	258	*gapA*
SRR5973279	258	258	*gapA*
SRR5973271	258	258	*gapA*
SRR5973336	258	258	*gapA*
SRR5973319	258	258	*gapA*
SRR5973317	258	258	*gapA*
SRR5973294	258	258	*gapA*
SRR5973291	258	258	*gapA*
SRR5973289	258	258	*gapA*
SRR5973400	258	258	*gapA*
SRR5973382	258	258	*gapA*
SRR5973381	258	258	*gapA*
SRR5973287	258	258	*gapA*
SRR5973240	307	307	*phoE*
SRR597324	307	307	*phoE*
SRR5973282	307	307	*phoE*
SRR5973280	307	307	*phoE*
SRR5973339	307	307	*phoE*
SRR5973322	307	307	*phoE*
SRR5973288	307	307	*phoE*
SRR5973396	307	307	*phoE*
SRR5973380	307	307	*phoE*
SRR5973379	307	307	*phoE*
SRR5973376	307	307	*phoE*
SRR5973373	307	307	*phoE*
SRR5973361	307	307	*phoE*
SRR5973355	307	307	*phoE*
SRR5973284	23	23	*phoE*
SRR5973332	35	35	*phoE*
SRR5973389	35	35	*phoE*
SRR5973368	35	35	*phoE*
SRR5973393	405	405	*phoE*
SRR5973311	412	412	*phoE*
SRR5973371	429	429	*tonB*
SRR5973327	466	466	*phoE*
SRR5973407	466	466	*phoE*
SRR5973239	492	492	*phoE*
SRR5973301	502	502	*phoE*
SRR5973348	753	753	*phoE*
SRR5973362	8	8	*phoE*

Our pipeline reported the presence of multiple variants at the same position for eight *E. coli* samples belonging to three different STs and 55 samples of *K. pneumonniae* belonging to 24 different STs (see Table 3). This might suggest the presence of contamination in the sequenced DNA samples or the existence of pseudogenes in the genome of the sampled organisms.

### Comparing BacTag with web-based tools for *E. coli* Achtman MLST

**Fig. 4:**
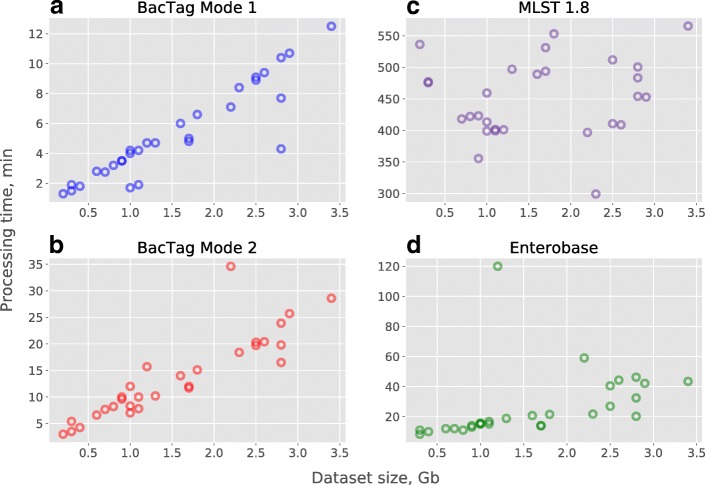
Time required for the analysis of 30 samples belonging to the ST131 by two modes of BacTag (**a** and **b**), MLST 1.8 (**c**) and Enterobase (**d**)

We measured the time required for the analysis, using 30 samples belonging to the *E. coli* ST131 with the dataset size varying from 0.2 to 3. Gb. We performed the MLST typing in two modes: with and without analysis of the low similarity sequences group. As can be seen in Fig. 4a and b, the processing time of BacTag depended on the sequencing sample size and the analysis mode. The larger the input sequencing data is, the more time is required for typing regardless of the analysis mode. Performing the typing including the analysis of low similarity group (mode 2) increases the processing time. Including low similarity sequences into the analysis did not affect the final output, for all samples tested during this research.

**Fig. 5:**
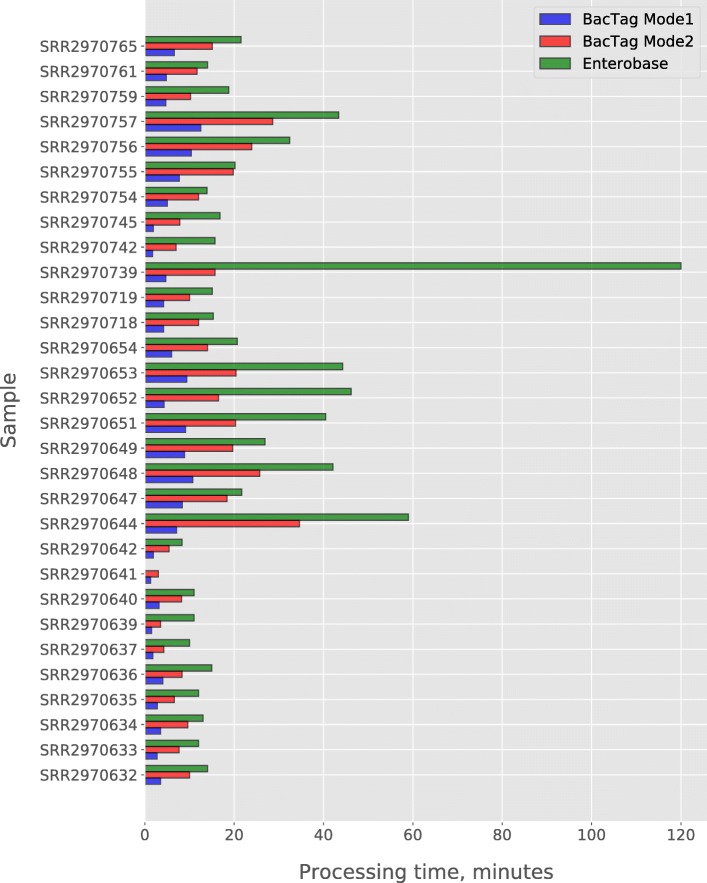
Comparing of the processing time required for the Achtman seven genes MLST analysis of 30 WGS *E. coli* samples

The same 30 samples were submitted for analysis to web-based tools for MLST typing: MLST1.8 [34] and Enterobase [35]. These methods perform the assembly of submitted WGS data and use the obtained contigs for the BLAST-based comparison with sequences in the MLST database. For both tools, the results of the WGS assembly can be downloaded after the analysis is finished, MLST 1.8 also provides information about BLAST alignments for the best matching alleles as an output. The analysis of the 30 samples with MLST 1.8 took from 299 to 569 (median 454) minutes per job, the processing time did not correlate with the input data size (Fig. 4c). MLST 1.8 failed to perform the assembly (and thus to finish the MLST) for two samples. Long processing time can be explained by high load of the tool server. However, that cannot be checked as it is only possible to track the time in between job submission to the server and the time when job is finished. It is unfortunately not possible to assess when the actual calculations for the particular sample started. Another tool, Enterobase, failed to perform the analysis of one sample (due to the problems with assembly) and did not define the correct ST for one other sample. However, Enterobase shows when each part of the analyzing pipeline is being launched, which allowed us to determine the time required for the analysis of each sample and compare it to our tool (Fig. 5). The processing time for Enterobase was comparable to our tool and also seems to be dependent on the size of the submitted WGS data (Fig. 4d).

## Discussions

In this paper we described BacTag – a new pipeline designed to perform fast and accurate gene and allele detection directly using WGS data. Our method was shown to work faster and more accurate than most popular current bioinformatics tools due to the absence of the necessity to compare sequencing data with each sequence in the database. Instead, we preprocess the reference database once prior to the analysis in order to store all the mismatches between different alleles of the same gene. Under the assumption that all alleles of the same gene are highly similar, it is easy to check whether the gene of interest is present in the sequencing data by mapping the reads to the most “average” gene allele. Variants detected after such mapping can be compared with the information obtained during the database preprocessing in order to retrieve the allele of the detected gene. Since the database preprocessing needs to be done only once, this approach significantly reduces the time required for the analysis of multiple samples. Additionally, the possibility of parallel computation allows to speed up the database preprocessing significantly since all of the independent computations can be done in parallel.

Most of the existing tools for automatic gene and allele detection are based on fixed and rarely updated databases. The possibility to choose the database that will be preprocessed as well as to check the quality of that database is another essential feature of BacTag. It is important to note that the pipeline allows the user to set the parameters for the database preprocessing and sequencing data analysis. The same database, preprocessed with different parameters, allows the user to control in which case the variants for some alleles are not properly called. Thus, the user can determine the optimal parameters to detect as many of the alleles of interest as possible and apply this knowledge to the experimental design. On the other hand, preprocessing the database with the parameters of already existing sequencing data provides an estimate of the alleles that likely will not be properly detected.

While the current tools for gene allele identification require assembly of the WGS data prior to the comparison with the reference database, we chose to work directly with raw sequencing data. This was done in order to preserve the information about positions with multiple reported variants, which would be lost in case of bacterial genome assembly. That information is crucial for the detection of possible sample contaminations, presence of pseudogenes and, potentially, for extending our pipeline to metagenomic datasets. Furthermore, BacTag can work with sequencing data that for some reasons cannot be assembled.

Two main limitations of the pipeline need to be addressed. First, our approach assumes that a considerable part of the same gene alleles is highly similar. The more alleles of the same gene that do not fulfill this requirement, the slower the pipeline will work: sequences for which the pipeline will not be able to call the proper variants will be checked by direct read mapping. Second, the pipeline also does not provide proper analysis results if several alleles of the same gene are present in the sequencing data (this can be caused, among other reason, by the mixed-strain infection of the same subject, see [62–64]). More detailed evaluation of the horizontal coverage of the detected genes as well as the additional analysis of the positions with multiple variants reported could potentially help to resolve this problem and extend the approach in order to perform the analysis on complicated metagenomic datasets.

## Conclusions

We have introduced BacTag – a new pipeline for fast and accurate gene and allele recognition based on database preprocessing and parallel computing. In contrast to the majority of already existing methods, BacTag avoids the comparison of sequencing data to each allele sequence present in the database due to the database preprocessing. While the database preprocessing provides analysis time reduction, it also provides important information about database quality. Amongst other advantages of our method are the possibility to cope with any user-provided database, and the absence of the assembly step that potentially may help extend our approach to metagenomics datasets. We believe that our approach can be useful for a wide range of projects, including bacterial subspecies classification, clinical diagnostics of bacterial infections, and epidemiological studies.

## Additional files

Additional file 1: BacTag dependencies. Programming languages and software packages used to build the BacTag pipeline. (PDF 23 kb)

Additional file 2: Flanking sequences. Flanking sequences used for the *E. coli*, *K. pneumoniae, S. pseudintermedius, P. gingivalis, M. bovis, Borrelia spp. and Streptomyces spp.* MLST databases preprocessing. (PDF 86 kb)

Additional file 3: Results of real sequencing data analysis. Summary of WGS *E. coli* and *K. pneumoniae* samples used for pipeline testing and the results of these tests. (PDF 97 kb)

## Abbreviations

MLST: Multi-locus sequence typing
NGS: Next-generation sequencing
ST: Sequence type
WGS: Whole-genome shotgun sequencing
