# Supplementary material for: BacTag - a pipeline for fast and accurate gene and allele typing in bacterial sequencing data based on database preprocessing
Source: BMC Genomics. 2019 May 6;20:338. doi: 10.1186/s12864-019-5723-0 (PMC6501397; doi:10.1186/s12864-019-5723-0)
Supplement: Supplementary file 1 — BacTag dependencies. Programming languages and software packages used to build the BacTag pipeline. (PDF 23 kb) [file 12864_2019_5723_MOESM1_ESM.pdf]

Programming language / Software	Version
Bash	4.3.11(1)
GNU Make	3.81
Python	3.5.1
Fastools	0.12.0
sim-reads	0.4.0
BWA	0.7.13-r1126
SAMtools	1.5
BCFtools	1.4
VCFtools	0.1.14

Table S1. Programming languages and software used in the pipeline
