# Supplementary material for: BacTag - a pipeline for fast and accurate gene and allele typing in bacterial sequencing data based on database preprocessing
Source: BMC Genomics. 2019 May 6;20:338. doi: 10.1186/s12864-019-5723-0 (PMC6501397; doi:10.1186/s12864-019-5723-0)
Supplement: Supplementary file 2 — Flanking sequences. Flanking sequences used for the E. coli, K. pneumoniae, S. pseudintermedius, P. gingivalis, M. bovis, Borrelia spp. and Streptomyces spp. MLST databases preprocessing. (PDF 86 kb) [file 12864_2019_5723_MOESM2_ESM.pdf]

| Gene | Flanking sequences |
| --- | --- |
| ADK | F ATTCTGCTTGGCGCTCCGGGNNN<br>R NNNNNAAATACGCGAAAGTTGACGG |
| FUMC | F TCCCGGCAGATAAGCTGTGGGGCGCACAAACTCAACGCTCGCTGGAGCATTTCCGCATTTCGACGGAGAAAATGCCCACCTCACTGATTCATGCGCTGGCGCTAACCAAGCGCGCAGCGGCAAAAGTTAATGAAGATTTAGGCTTGTTGTCTGAAGAGAAAG<br>R TATGGGCGGTACAGCTGTGGGTACTGGACTAAATACCCATCCGGAGTATGCGCGTCGCGTGGCAGATGAACTGGCAGTCATTACCTGCGCACCGTTTGTTACCGCGCCGAACAAATTTGAAGCGCTGGCGACCTGTGA |
| GYRB | F TCGGCGACACGGATGACGGCACCGGTCTGCACCACTGGTATTCGAGGTGGTAGATAACGCTATCGACGAAGCGCTCGCGGGTCACTGTAAAGAAATTATCGTCACCATTCACGCCGACAACTCTGTCTCTGTACAGGATGACGGGCGCGGCATTCCGACCGGTATTCACCCGGAAGAGGGCGTATCGGCGGCGGAAGTGATCATGACCGTTCTGCACGCAGGCGGTAAATTTGACGATAACTCCTATAAAGTGTCCGGC<br>R ACTGCTTTACCAACAACATTCCACAGCGTGACGGCGGTACTCACCTGGCAGGCTTCCGTGCGGCAATGACCCGTACCCTGAACGCCTACATGGAC |
| ICD | F ATGGAAAGTAAAGTAGTTGTTCCGGCACAAGGCAAGAAGATCACCCTGCAAAACGGCAAACTCAACGTTCCTGAAAATCCGATTATCCCTTACATTGAAGGTGATGGAATCGGTGTAGATGTAACCCCAGCCATGCTGAAAGTGGT<br>R GTGACTCTGTGACCCTGGTGCACAAAGGCAACATCATGAAGTTCACCGAAGGCGCGTTTAAAGACTGGGGCTACCAGCTGGCGCGTGAAGAGTTTGGCGGTGAACTGATCGACGGCGGCCCGTGGCTGAAAGTTAAAAACCCGAATACCGGCAAAGAGATCGTCATTAAAGATGTGATTGCTGATGCATTCCTGCAACAGATCCTGCTGCGTCC |
| MDH | F CAGGTTCAGAACTCTCTCTGTATGATATCGCTCCAGTGACTCCCGGTGTGGCTGTCGATCTGAGCCATATCCCTACTGCTGTGAAAATCAAAGGTTTTTCTGGTGAAGATGCGACTCCGGCGCTGGAAGGCGCAAATGTCGTTCTTATCTCTGCA<br>R CCAGGCAGCTGCACGTTTTGGTCTGTCTCTGGTACGCGCACTGCAGGGCGAACAAGGCGTTGTCGAATGTGCCTACGTTGAAGGCGACGGTCAGTACGCCCGTTTCTTCTCTCAACCGCTGCTGCTGGGTAAAAACGGCGTGGAAGAGCGTAAATCTATCGGCACTCTGAGCGCATTTGAAAAGAACGCGCT |
| PURA | F CGCGCTGATGAAAGAGATGAAAGAACTGGAAGACCGTGGCATCCCCGTTCGTGAGCGTCTGCTGCTG TCTGAAGCATGTCCGCTGATCCTTGATTATCACGTTGCGCTGG<br>R GGGTGCAGGTCCGTTCCCGACTGAACTGTTTGATGAAACTGGCGAGTTCCTCTGCAAGCAGGGTAACGAATTCGGCGCAACTACGGGTCGTCGTCGTCGTACCGGCTGGCTGGACACCGTTGCCGTTCGTCGTGCGGTACAGCTGAACTCCCTGTCTGGCTTCTGCCTGACCAAGCTGGACGTTCTGGATGGCCTGAAAGAGGTGAAACTCTGCGTGGCTTACCGTATG |
| RECA | F AGCGTGAAGGTAAAACCTGTGCGTTTATCGATGCTGAACACGCGCTGGACCCAATCTATG<br>R ACTGGTTGATCTGGGCGTGAAAGAGAAGCTGATCGAGAAAGCAGGTGCGTGGTACAGCTACAAAGGT |

Table S2. Flanking sequences used for the *E. coli* MLST database preprocessing. F – forward, R – reverse.

| Gene | Flanking sequences |
|---|---|
| GAPA | F TGAAGTATGACTCCACTCACGGTCGTTTCGACGGTACCGTTGAAGTGAAAGACGGTCATCTGGTCGTTAACGGTAAAAAAATCCGTGTTACCGCTGAACGTGACCCGGCT<br>R CGCGTTCCGACTCCGAACGTATCTGTTGTTGACCTGACCGTTCGTCTGGAAAAAGCAGCGTCCTACGAAGAAATCAAGAAAGCCATCAAAGCCGCTTCTGAAG |
| INFB | F CTCGCTGCTGGACTACATTCGTTCTACCAAGGTTGCCTCCGGCGAAGCGGGTGGATTACCCAGCACATCGGTGCTTACCACGTCGAAACCGACAAC<br>R CTGCTGGACGCGATCCTGCTGCAGGCTGAAGTTCTTGAGCTGAAAGCG |
| MDH | F CCCAACTGCCTTCAGGTTCAGAGCTCTCGTTGTACGACATCGCTCCGGTTACACCGGGCGTGGCGGTAGATCTAAGTCATATCCCCACAGATGTAAAAATTAAAGGATTTTCCGGTGAAGACGCTACTCCGGCGCTGGAA<br>R GGCCAGGCGGCTGCCCGTTTTGGTCTCTCTCTGGTTCGCGCCATGCAGGGGGAAAAAGG |
| PGI | F GAGAAAAACCTGCCGGTGCTGCTGGCGCTGATCGGCATCTGGTACAACAACTTCTTCGGTGCGGAAACCGAAGCGATTCTGCCGTACGACCAGTACATGCACCGCTTTGCCGCTTACTTCCAGCAGGGCAACATG<br>R ATCCTCAACATCTTCACCTTTGACCAGTGGGGCGTTGAGCTGGGCAAACAGCTGGCTAACCGCATCCTGCCGGAGCTGAAAGACGGCAGCGAAGTTAGCAGCCACGACAGCTCTACTAACGGCCTGATTAACCGTTATAAAGCCTGGCGCG |
| PHOE | F ACCTACCGCAACACCGACTTCTTCGGTCTGGTGGATGGCCTGGATCTGACCCTGCAGTACCAGGGTAAAAACGAAGGCCGTGAAGCGAAGAAACAGAACGGCGACGGCG<br>R CTGAAAAGCGATAACAAACTCGGCATCAACGATGACGACATCGTCGCGCTGGGTATGACCTACCAGTTCTGA |
| RPOB | F GGCGAAATGGCGGAAAACCAGTTCCGCGTTGGCCTGGTACGTGTAGAGCGTGCGGTGAAAGAGCGTCTGTCTCTTGGCGATCTGGATACCCTGATGCCTCAGGATATGATCAACGCCAAGCCGATTTCCGCAGCAGTGAAAGAGTTCTTTGGTTCCAGCCAGCTGTCTCAGTTTATGGACCAGAACAACCCGCTGTCTGAGATTACGCACAAACGTCGTATCTCCGCACTCGGCCCAGGCGGTCTGACCCGTGAGCGCGCAGGCTTCGAAGTTCGAGACGTACACCCG<br>R CGTGGCGGTACCGTTCAGTACGTGGATGCTTCCCGTATCGTTATCAAAGTTAACGAAGACGAGATGTACCCGGGCGAAGCAGGTATCGACATCTATAACCTGACCAAGTACACCCGTTCTAACCAGAATACCTGCATCAACCAGATGCCTTGCGTGTCCCTGGGCGAACCTATTGAGCGCGGCGACGTGCTGGCAGACGGCCCGTCCACCGACCTCGGTGAGCTGGCGCTGGGTCAGAACATGCGTGTAGCGTTCATGCCGTGGAACGGTTACAACTTCGAAGACTC |
| TONB | F CTTTATACCTCGGTACATCAGGTTATTGAACAGCCTTCTCCGACGCAGCCGATAGAGATCACA<br>R TTTGACGTTTCGCCTGATGGCCGCATTGATAATCTGCAGATCCTCTCTGCTCAGCCGGCGAAT |

Table S3. Flanking sequences used for the *K. pneumoniae* MLST database preprocessing. F – forward, R – reverse.

| Gene | Flanking sequences |
|---|---|
| ACK | F CACCACTTCACAACCCAGCAAACTTAATGGGAATTCAAGCATTTAGAAAATTGCTTCCTGAAA<br>R TGCCGGTGTAGGTGAAAACTCAGATGCAGTGCGTGCGCGTGTATTAGAAGGTT |
| CPN60 | F GCGACTGTACTTGCACAAGCAATGATTCAAGAAGGACTTAAAAACGTGACAAGTGGTGCGA<br>R ATGCGTTAACAAACCTTGTCTTAAACCGTATGCGTGGTACATTTACAGCGGTTGCAGTT |
| PTA | F GTGCGTATCGTATTACCAGAAGGAGAAGATGAGCGTGTATTA<br>R GACCCACGTGTAGCGATGTTAAGCTTCTCAACAAAAGGTTCTGC |
| FDH | F CTTCTCATGATTCACCGGCAAACAAAATTTCTTCATGTGTGAAAGGGAAATTCGGTTGGGATTACGTCAA TTCAGAAGAGCGTTTAACGAAACCATTAATCCGCCGTGGTGATCAATT<br>R GATATCGGTAAAGCAGAAATGGTTATTACGATCGGTACGAATACAGCAGAAGCACATCCTGTTATCGCA |
| PURA | F GATTACTTCCAAGGTATGTTTAAACAAGATGCACCATCATTTGAAGACATTTTTGAAACATAC<br>R GACTTATCTATTAACTCTATCGA |
| SAR | F GGATTTAGTCCAGTTCAAAATTTAACACAAGCATTAGGCAATCCTCAAAGTGGCGGTGCGG<br>R TAAAACCGCTGCTGCTCAAAATATCGTAGCAATTAACTTTATTAATGATCAACTCGGTCATGACGTTTCAT GGGGCGAATGGTTC |
| TUF | F GCTTCAGCGTAGTCTAATAACTTACGGAACATTTCTACACCAGTAAC<br>R CGAGTTTGTGGCATTG |

Table S4. Flanking sequences used for the *S. pseudintermediuы* MLST database preprocessing. F – forward, R – reverse.

| Gene | Flanking sequences |
|---|---|
| ADH1 | F TGCTAGTTGTTCAAACACGTCTTTTACTTCATCTAAGCTAATAATTCTTCCAAT<br>R TTGCAACATAGTCTCCAACTTTTAATTCAGCTTGTTTAAGTGCTTTGTAACTAGTTACTCC |
| GLTX | F GTTTTGAGAATCATTGCATTTTTTTAATGTCAAATTTTGAAGGAGACTTACT<br>R ATCTCATTCATAAGTAGTATCCTTAGGCATACAAAACCTTATTGAATACTCACCA |
| GPSA | F CCAATTCCAATTGCTAAAACATTTTTAAGTGCAGCAAATAATTCACTACCTTT<br>R ATTTATGCCACTATTAATATCAGATATTTCTTTTTGATCAATTCCTCACATTTT |
| GYRB | F AGCTTGCTAATTGCACCAGCAAAAATGCTGAAATCTGTGAATTATACATCGTT<br>R GATGTTTTGCCAAGAAGAGAATTTATTGAAAAAAATGCAAAATTTGTTCAAAATA |
| PTA2 | F CTTAGCTTTTCTTACATTTAGGTCATAGGCTGAATCAAATTGCATTTCACCATTAAT<br>R TCTGGTGTGCATATTTTTTAAGCAAGTTTCATAGTCTTCTTTGCCATTACGAATT |
| TDK | F TATCTCATAGCTTTTTTAGCTTCGCCTTTGATGTGACAAACTCTGCATCTAGCTTCATATTCAGAATCGCCT<br>AATAAATTAAGCTCATCGCTCTTAACTTTTCTAAAAGAAAATCCTGCATCAGATTTGCATTCTAAACAAACAG<br>CTTTAAGCTTTAAAATGTTATCAGCGGAAGCTAAAACTCTAGCCATCACATCAAATGGTCTTCT<br>R GGAAAACATTGGTCCAGTTATAACCTCTAATTGACCTAATCCACTTTTTAAATACAT |
| TKT | F CCACCATATAAATTAATGCCATTATTAATTGCAGTCATTGCAAATTCTCTTA<br>R GTTTTCAAATGGAGTAAATGGGGCTATTATATTGTGCACCATAATATAAGTTGG |

Table S5. Flanking sequences used for the M. bovis MLST database preprocessing. F – forward, R – reverse.

| Gene | Flanking sequences |
|---|---|
| FTSQ | F TTGCAGCCATAGTTTACTTTTCGGACTATTCCGATAGCACTCGCTGCTCAGGTTTGGAAGTTCGAGTCGA GGGTAAGAC<br>R CATATTAGTTCCCCGTCTGGGCAAAACTGAAATTATCATAGGCAGTACACCTAATTGGGCAGAAAAA |
| GPDXJ | F GGCAATGTCCCCGATGTCCTCCGAGTGGCTCTCGACTGTGAGCGGTTCGGTGCACAG<br>R GGCTGCTGCCGTACAGCCCTATGTCAAGGCTTCGGCACACGCTCATTCGCTCGGTCTCGGGTATC |
| HAGB | F CGTCTGCAGCGGCTCCAAAATTTGGAGCACTACCGTTTTGCCAAGAATGTGCTGACGCTCTGTCGCACG GCAAATATCGCTAAACTGAATCCCA<br>R CGTCTGAGCACCGACCAACGAGGCAAATATGACGTGAAGGCACTCCGTGCCGAGACCGACCGCACATT GGTAGCCGTGGTGCGCCG |
| MCMA | F TGCCCGTCCTGTCCCATTTTGGCGATCATGATACGCGGCTGACGGCCTTCTTTCTTTGCAAACTTCTCTGC CAA<br>R TCCGGTCTCGATGGCTTTTGCCATTCCGCCCATCTCCTGTACTTCCTTGATGAGTGTCCACGCCTTGT |
| PEPO | F AGCTGCTTACCATACCTACGGAACGTTTCCAGCGAGGATGCATTTCGGTGGTTCCACTCAGCGTCTTGCC GAAAAAGTCGAAACGGGCTTGCTCGAAGTCATCGCTCAGATAGCTTGCTGCACCGCTGATCGT<br>R AAGCCAGTCTGGGTGATATGGAAGATGTTCATCTCGCTATTCTTGGCGTCGGCATAAACGTACGATCCG AAGAATGTGCTTCCACCCAT |
| PGA | F GATCTGCCCTGCCAACCACACACCACCATCCCTCTTCGCAAGTAATCGACCTGCGCGACAAACTCTACATGA TCGATTGCGGAGAAGGTGTCCAGCGACAATTCAGACACGAAAAGCTCCATTTCGGACGT<br>R ATGGGCGGATCATCCCCAACCGACACCTCACGACCCCGGGTACACCACCCAGACGATATGCTTACTGCTC GGACACGGAGTTTTGTCCCTCCATCGTCCCTATTATTCAAGGTGTGGATTTGCTCTACCATGAAGCTACTTTT ATGGAGGAGGATTGGGCAA |
| RECA | F GAATGTCGATGCGGATGGATGCATAGAATTTCAGTGCATTCCCCCCTGTGGTGGTCTCCGGATTGCCGA ACAAAACACCGATCTTCTCTCGTAGCTGGTTGATGAAGATACAGG<br>R GCCTACACCGAGAGCCAAATCCAAACCGATGGAGCCGGAGGGGATAACGCTTACATCCTCCACCGTATT GGCTCCCATATTCATGATAGCACCTTTTCCGAATGTCTTCTCTATCTTCTCCGTGGCCA |

Table S6. Flanking sequences used for the P. *gingivalies* MLST database preprocessing. F – forward, R – reverse.

| Gene | Flanking sequences |
| --- | --- |
| CLPA | F TTCATCTCTTCAATAATAGCCTTTTCAAAATCACTCTCACCTACAGGCCT<br>R TATTTTATGCTCTAAATTAATCATTAAGTCTTGATCATAAGCATCAAAAT |
| CLPX | F ATGAGATGAATGTTCCGTTTGCAATAGCGGATGCTACAACGCTTACTGAG<br>R ATGTTAAAAAATACAGGTGCTAGAGGTTTGCGTTCTATTTTAGAAGAATT |
| NIFS | F ATGAAAAATTAAAAATCATAAGGAAAGATTTTCCTATTCTAAATAAAACT<br>R AACGATTGGAAAGATTGCTAGAGATAACAAAATTATTCTGTTCATTGATG |
| PEPX | F ATTGTGGTTCCGGCATTGGTATTTGCTTTCATTATTATTTCTTGAATTTT<br>R TGCAAATCCTTCATTTGCCTTTCGGTTTAAGTGAATAGCGACATTTGGTA |
| PYRB | F TGGAAAATATTTTGTTTATTGAAACAATGAGACAGATAAGATATGAGATT<br>R AAATATGCACGGGAGAATAATATTCCTTTTCTTGGCATTTGTCTTGGCAT |
| RECG | F TAATTGAAGCTGGATATCAAGTTGCGTTGATGGTGCCTACTGATCTTTTG<br>R GTTATTGAAGTTGGTATTGATTGCCCAAATGCGACTTGTATGGTAGTAG |
| RPLB | F CTTCATCTTTGCGTTATAAGACAACTTTATCATTTGATGAGTTGAGTAAG<br>R GGAGGTGGTGAGGGTAAGACTTCTGGTGGTCGTCATCCTGTATCACCTTG |
| UVRA | F TGAAATTTTTAATTGATGTTGGACTTTCTTATTTATATTTAGATAGAATG<br>R ATCATTGGGTATGATCAAATTGATAAGGTTATTCAGATAAACCAAAAACC |

Table S7. Flanking sequences used for the *Borrelia spp.* MLST database preprocessing. F – forward, R – reverse.

| Gene | Flanking sequences |
|---|---|
| ATPD | F TCGACGAGCAGTACACCGGTGAGCGCTGGCCGATCCACCGCAAGGCCCCG |
| | R CAGGAGCGCATCACCTCGACCCGTGGTCACTCGATCACCTCGATGCAGGC |
| GYRB | F TTGTCCTCGGCCTCGAGGTCGATCACGGTGGGGTGCACCAGCTCTCCCTT |
| | R ACCACCGGAGACCGCGTAACCGCCGCCGCCGAACTTGCCGCCCGCGTGCA |
| RECA | F ACCGGACCAACGAGCCCATCGAGGTCATCCCGACCGGGTCTACCGCGCTC |
| | R AAGTTCTACGCCTCGGTGCGACTCGACATCCGGCGGATCGAGACGCTGAA |
| RPOB | F ACGTGCACCCGTCGCACTACGGCCGCATGTGCCCGATCGAGACGCCCGAA |
| | R CGCCTGGCCAAGTTCTCCCGGTCCAACCAGGGCACCTCGGTCAACCAGAA |
| TRPB | F GAGTCCTTGAGGTAGGCGTGCTCCGGGCCGATGCCGGGGTAGTCCAGACC |
| | R GCCCGCGCCGGTCTCGGCGATGACGCGGGTCTTGCCCATGCGCTTGGTGA |

Table S8. Flanking sequences used for the *Streptomyces spp.* MLST database preprocessing. F – forward, R – reverse.
