# Supplementary material for: BacTag - a pipeline for fast and accurate gene and allele typing in bacterial sequencing data based on database preprocessing
Source: BMC Genomics. 2019 May 6;20:338. doi: 10.1186/s12864-019-5723-0 (PMC6501397; doi:10.1186/s12864-019-5723-0)
Supplement: Supplementary file 3 — Results of real sequencing data analysis. Summary of WGS E. coli and K. pneumoniae samples used for pipeline testing and the results of these tests. (PDF 97 kb) [file 12864_2019_5723_MOESM3_ESM.pdf]

Samples Metadata		Experiments results		Reported closest allele pere housekeeping gene						
BioProject	Run	Expected ST	Reported ST	ADK	FUMC	GYRB	ICD	MDH	PURA	RECA
PRJEB12510	ERR1227184	101	101	43	41	15	18	11	7	6
PRJEB6308	ERR556934	127	127	13	14	19	36	23	11	10
PRJEB6308	ERR556935	127	127	13	14	19	36	23	11	10
PRJNA297860	SRR2970632	131	131	53	40	47	13	16	28	29
PRJNA297860	SRR2970633	131	131	53	40	47	13	16	28	29
PRJNA297860	SRR2970634	131	131	53	40	47	13	16	28	29
PRJNA297860	SRR2970635	131	131	53	40	47	13	16	28	29
PRJNA297860	SRR2970636	131	131	53	40	47	13	16	28	29
PRJNA297860	SRR2970637	131	131	53	40	47	13	16	28	29
PRJNA297860	SRR2970639	131	131	53	40	47	13	16	28	29
PRJNA297860	SRR2970640	131	131	53	40	47	13	16	28	29
PRJNA297860	SRR2970641	131	131	53	40	47	13	16	28	29
PRJNA297860	SRR2970642	131	131	53	40	47	13	16	28	29
PRJNA297860	SRR2970643	131	131	53	40	47	13	16	28	29
PRJNA297860	SRR2970644	131	131	53	40	47	13	16	28	29
PRJNA297860	SRR2970645	131	131	53	40	47	13	16	28	29
PRJNA297860	SRR2970646	131	131	53	40	47	13	16	28	29
PRJNA297860	SRR2970647	131	131	53	40	47	13	16	28	29
PRJNA297860	SRR2970648	131	131	53	40	47	13	16	28	29
PRJNA297860	SRR2970649	131	131	53	40	47	13	16	28	29
PRJNA297860	SRR2970650	131	131	53	40	47	13	16	28	29
PRJNA297860	SRR2970651	131	131	53	40	47	13	16	28	29
PRJNA297860	SRR2970652	131	131	53	40	47	13	16	28	29
PRJNA297860	SRR2970653	131	131	53	40	47	13	16	28	29
PRJNA297860	SRR2970654	131	131	53	40	47	13	16	28	29
PRJNA297860	SRR2970655	131	131	53	40	47	13	16	28	29
PRJNA297860	SRR2970657	131	131	53	40	47	13	16	28	29
PRJNA297860	SRR2970668	131	131	53	40	47	13	16	28	29
PRJNA297860	SRR2970680	131	131	53	40	47	13	16	28	29
PRJNA297860	SRR2970690	131	131	53	40	47	13	16	28	29

PRJNA297860	SRR2970691	131	131	53	40	47	13	16	28	29
PRJNA297860	SRR2970692	131	131	53	40	47	13	16	28	29
PRJNA297860	SRR2970693	131	131	53	40	47	13	16	28	29
PRJNA297860	SRR2970694	131	131	53	40	47	13	16	28	29
PRJNA297860	SRR2970695	131	131	53	40	47	13	16	28	29
PRJNA297860	SRR2970696	131	131	53	40	47	13	16	28	29
PRJNA297860	SRR2970697	131	131	53	40	47	13	16	28	29
PRJNA297860	SRR2970698	131	131	53	40	47	13	16	28	29
PRJNA297860	SRR2970699	131	131	53	40	47	13	16	28	29
PRJNA297860	SRR2970700	131	131	53	40	47	13	16	28	29
PRJNA297860	SRR2970701	131	131	53	40	47	13	16	28	29
PRJNA297860	SRR2970702	131	131	53	40	47	13	16	28	29
PRJNA297860	SRR2970703	131	131	53	40	47	13	16	28	29
PRJNA297860	SRR2970704	131	131	53	40	47	13	16	28	29
PRJNA297860	SRR2970705	131	131	53	40	47	13	16	28	29
PRJNA297860	SRR2970706	131	131	53	40	47	13	16	28	29
PRJNA297860	SRR2970707	131	131	53	40	47	13	16	28	29
PRJNA297860	SRR2970708	131	131	53	40	47	13	16	28	29
PRJNA297860	SRR2970709	131	131	53	40	47	13	16	28	29
PRJNA297860	SRR2970710	131	131	53	40	47	13	16	28	29
PRJNA297860	SRR2970711	131	131	53	40	47	13	16	28	29
PRJNA297860	SRR2970712	131	131	53	40	47	13	16	28	29
PRJNA297860	SRR2970713	131	131	53	40	47	13	16	28	29
PRJNA297860	SRR2970714	131	131	53	40	47	13	16	28	29
PRJNA297860	SRR2970715	131	131	53	40	47	13	16	28	29
PRJNA297860	SRR2970716	131	131	53	40	47	13	16	28	29
PRJNA297860	SRR2970717	131	131	53	40	47	13	16	28	29
PRJNA297860	SRR2970718	131	131	53	40	47	13	16	28	29
PRJNA297860	SRR2970719	131	131	53	40	47	13	16	28	29
PRJNA297860	SRR2970720	131	131	53	40	47	13	16	28	29
PRJNA297860	SRR2970728	131	131	53	40	47	13	16	28	29
PRJNA297860	SRR2970733	131	131	53	40	47	13	16	28	29

PRJNA297860	SRR2970734	131	131	53	40	47	13	16	28	29
PRJNA297860	SRR2970735	131	131	53	40	47	13	16	28	29
PRJNA297860	SRR2970737	131	131	53	40	47	13	16	28	29
PRJNA297860	SRR2970738	131	131	53	40	47	13	16	28	29
PRJNA297860	SRR2970739	131	131	53	40	47	13	16	28	29
PRJNA297860	SRR2970740	131	131	53	40	47	13	16	28	29
PRJNA297860	SRR2970741	131	131	53	40	47	13	16	28	29
PRJNA297860	SRR2970742	131	131	53	40	47	13	16	28	29
PRJNA297860	SRR2970743	131	131	53	40	47	13	16	28	29
PRJNA297860	SRR2970744	131	131	53	40	47	13	16	28	29
PRJNA297860	SRR2970745	131	131	53	40	47	13	16	28	29
PRJNA297860	SRR2970746	131	131	53	40	47	13	16	28	29
PRJNA297860	SRR2970747	131	131	53	40	47	13	16	28	29
PRJNA297860	SRR2970748	131	131	53	40	47	13	16	28	29
PRJNA297860	SRR2970749	131	131	53	40	47	13	16	28	29
PRJNA297860	SRR2970750	131	131	53	40	47	13	16	28	29
PRJNA297860	SRR2970751	131	131	53	40	47	13	16	28	29
PRJNA297860	SRR2970752	131	131	53	40	47	13	16	28	29
PRJNA297860	SRR2970753	131	131	53	40	47	13	16	28	29
PRJNA297860	SRR2970754	131	131	53	40	47	13	16	28	29
PRJNA297860	SRR2970755	131	131	53	40	47	13	16	28	29
PRJNA297860	SRR2970756	131	131	53	40	47	13	16	28	29
PRJNA297860	SRR2970757	131	131	53	40	47	13	16	28	29
PRJNA297860	SRR2970758	131	131	53	40	47	13	16	28	29
PRJNA297860	SRR2970759	131	131	53	40	47	13	16	28	29
PRJNA297860	SRR2970760	131	131	53	40	47	13	16	28	29
PRJNA297860	SRR2970761	131	131	53	40	47	13	16	28	29
PRJNA297860	SRR2970762	131	131	53	40	47	13	16	28	29
PRJNA297860	SRR2970763	131	131	53	40	47	13	16	28	29
PRJNA297860	SRR2970764	131	131	53	40	47	13	16	28	29
PRJNA297860	SRR2970765	131	131	53	40	47	13	16	28	29
PRJNA297860	SRR2970766	131	131	53	40	47	13	16	28	29

PRJNA297860	SRR2970767	131	131	53	40	47	13	16	28	29
PRJNA297860	SRR2970768	131	131	53	40	47	13	16	28	29
PRJNA297860	SRR2970769	131	131	53	40	47	13	16	28	29
PRJNA297860	SRR2970770	131	131	53	40	47	13	16	28	29
PRJNA297860	SRR2970771	131	131	53	40	47	13	16	28	29
PRJNA297860	SRR2970772	131	131	53	40	47	13	16	28	29
PRJNA297860	SRR2970773	131	131	53	40	47	13	16	28	29
PRJNA297860	SRR2970774	131	131	53	40	47	13	16	28	29
PRJNA297860	SRR2970775	131	131	53	40	47	13	16	28	29
PRJNA297860	SRR2970776	131	131	53	40	47	13	16	28	29
PRJNA297860	SRR2970777	131	131	53	40	47	13	16	28	29
PRJNA297860	SRR2970778	131	131	53	40	47	13	16	28	29
PRJNA297860	SRR2970779	131	131	53	40	47	13	16	28	29
PRJNA297860	SRR2970780	131	131	53	40	47	13	16	28	29
PRJNA297860	SRR2970781	131	131	53	40	47	13	16	28	29
PRJNA297860	SRR2970782	131	131	53	40	47	13	16	28	29
PRJNA297860	SRR2970783	131	131	53	40	47	13	16	28	29
PRJNA298688	SRR2767732	16	16	6	4	12	16	9	7	12
PRJNA298688	SRR2767734	21	21	16	4	12	16	9	7	7
PRJEB12765	ERR1276256	38	38	4	26	2	25	5	5	19
PRJEB12765	ERR1276257	38	38	4	26	2	25	5	5	19
PRJEB12765	ERR1276258	38	38	4	26	2	25	5	5	19
PRJEB12765	ERR1276259	38	38	4	26	2	25	5	5	19
PRJEB12765	ERR1276260	38	38	4	26	2	25	5	5	19
PRJEB12765	ERR1276261	38	38	4	26	2	25	5	5	19
PRJEB12765	ERR1276262	38	38	4	26	2	25	5	5	19
PRJEB12765	ERR1276263	38	38	4	26	2	25	5	5	19
PRJEB12765	ERR1276264	38	38	4	26	2	25	5	5	19
PRJEB12765	ERR1276265	38	38	4	26	2	25	5	5	19
PRJEB12765	ERR1276266	38	38	4	26	2	25	5	5	19
PRJEB12765	ERR1276267	38	38	4	26	2	25	5	5	19
PRJEB12765	ERR1276268	38	38	4	26	2	25	5	5	19

PRJEB12765	ERR1276269	38	38	4	26	2	25	5	5	19
PRJEB12765	ERR1276270	38	38	4	26	2	25	5	5	19
PRJEB12765	ERR1276271	38	38	4	26	2	25	5	5	19
PRJEB12765	ERR1276272	38	38	4	26	2	25	5	5	19
PRJEB12765	ERR1276273	38	38	4	26	2	25	5	5	19
PRJEB12765	ERR1276274	38	38	4	26	2	25	5	5	19
PRJEB12765	ERR1276275	38	38	4	26	2	25	5	5	19
PRJEB12765	ERR1276276	38	38	4	26	2	25	5	5	19
PRJEB12765	ERR1276277	38	38	4	26	2	25	5	5	19
PRJEB12765	ERR1276278	38	38	4	26	2	25	5	5	19
PRJEB9568	ERR1197942	410	410	6	4	12	1	20	18	7
PRJEB9568	ERR1197943	410	410	6	4	12	1	20	18	7
PRJEB9568	ERR1197944	410	410	6	4	12	1	20	18	7
PRJEB9568	ERR1197945	410	410	6	4	12	1	20	18	7
PRJEB9568	ERR1197946	410	410	6	4	12	1	20	18	7
PRJEB9568	ERR1197947	410	410	6	4	12	1	20	18	7
PRJEB9568	ERR1197948	410	410	6	4	12	1	20	18	7
PRJEB9568	ERR1197949	410	410	6	4	12	1	20	18	7
PRJEB9568	ERR1197950	410	410	6	4	12	1	20	18	7
PRJEB9568	ERR1197951	410	410	6	4	12	1	20	18	7
PRJEB9568	ERR1197952	410	410	6	4	12	1	20	18	7
PRJEB9568	ERR1197953	410	410	6	4	12	1	20	18	7
PRJEB9568	ERR1197954	410	410	6	4	12	1	20	18	7
PRJEB9568	ERR1197955	410	410	6	4	12	1	20	18	7
PRJEB9568	ERR1197956	410	410	6	4	12	1	20	18	7
PRJEB9568	ERR1197957	410	410	6	4	12	1	20	18	7
PRJEB9568	ERR1197958	410	410	6	4	12	1	20	18	7
PRJEB9568	ERR1197959	410	410	6	4	12	1	20	18	7
PRJEB9568	ERR1197960	410	410	6	4	12	1	20	18	7
PRJEB9568	ERR1197961	410	410	6	4	12	1	20	18	7
PRJEB9568	ERR1197962	410	410	6	4	12	1	20	18	7
PRJEB9568	ERR1197963	410	410	6	4	12	1	20	18	7

PRJEB9568	ERR1197964	410	410	6	4	12	1	20	18	7
PRJEB9568	ERR1197965	410	410	6	4	12	1	20	18	7
PRJEB9568	ERR1197966	410	410	6	4	12	1	20	18	7
PRJEB9568	ERR1197967	410	410	6	4	12	1	20	18	7
PRJEB9568	ERR1197968	410	410	6	4	12	1	20	18	7
PRJEB9931	ERR966595	73	73	36	24	9	13	17	11	25
PRJEB9931	ERR966596	73	73	36	24	9	13	17	11	25
PRJEB9931	ERR966597	73	73	36	24	9	13	17	11	25
PRJEB9931	ERR966598	73	73	36	24	9	13	17	11	25
PRJEB9931	ERR966599	73	73	36	24	9	13	17	11	25
PRJEB9931	ERR966600	73	73	36	24	9	13	17	11	25
PRJEB9931	ERR966601	73	73	36	24	9	13	17	11	25
PRJEB9931	ERR966602	73	73	36	24	9	13	17	11	25
PRJEB9931	ERR966603	73	73	36	24	9	13	17	11	25
PRJEB9931	ERR966604	73	95	37	38	19	37	17	11	26
PRJEB9931	ERR966605	73	73	36	24	9	13	17	11	25
PRJEB9931	ERR966606	73	73	36	24	9	13	17	11	25
PRJEB9931	ERR966607	73	73	36	24	9	13	17	11	25
PRJEB9931	ERR966608	73	73	36	24	9	13	17	11	25
PRJEB9931	ERR966609	73	73	36	24	9	13	17	11	25
PRJEB9931	ERR966610	73	73	36	24	9	13	17	11	25
PRJEB9931	ERR966611	73	73	36	24	9	13	17	11	25
PRJEB9931	ERR966612	73	73	36	24	9	13	17	11	25
PRJEB9931	ERR966613	73	73	36	24	9	13	17	11	25
PRJEB9931	ERR966614	73	73	36	24	9	13	17	11	25
PRJEB9931	ERR966615	73	73	36	24	9	13	17	11	25
PRJEB9931	ERR966616	73	73	36	24	9	13	17	11	25
PRJNA30551	SRR001666	92	92	10	11	4	8	8	8	2

Table S9. The summary of WGS *E. coli* samples used for pipeline testing and results of testing

(green)	non-homozygous sites detected
(orange)	predicted and expected ST do not match
(light yellow)	variants to not match completely with closest allele

Samples Metadata		Experiments results		Reported closest allele pere housekeeping gene						
BioProject	Run	Expected ST	Reported ST	GAPA	INFB	MDH	PGI	PHOE	RPOB	TONB
PRJNA396774	SRR5973323	101	101	2	6	1	5	4	1	6
PRJNA396775	SRR5973350	101	101	2	6	1	5	4	1	6
PRJNA396777	SRR5973260	111	111	2	1	5	1	17	4	42
PRJNA396778	SRR5973405	1164	1164	2	2	1	1	1	1	211
PRJNA396779	SRR5973308	1180	1180	2	1	2	1	1	1	34
PRJNA396780	SRR5973303	13	13	2	3	1	1	10	1	19
PRJNA396781	SRR5973253	133	133	12	1	1	2	5	1	36
PRJNA396783	SRR5973334	133	133	12	1	1	2	5	1	36
PRJNA396785	SRR5973324	1373	1373	2	1	1	1	1	4	4
PRJNA396786	SRR5973298	1412	1412	2	5	1	1	4	1	18
PRJNA396787	SRR5973251	1426	1426	3	5	1	1	12	4	46
PRJNA396788	SRR5973269	147	147	3	4	6	1	7	4	38
PRJNA396789	SRR5973310	1496	1496	2	1	1	37	3	4	64
PRJNA396790	SRR5973249	15	15	1	1	1	1	1	1	1
PRJNA396791	SRR5973270	15	15	1	1	1	1	1	1	1
PRJNA396792	SRR5973395	16	16	2	1	2	1	4	4	4
PRJNA396793	SRR5973378	16	16	2	1	2	1	4	4	4
PRJNA396794	SRR5973262	17	17	2	1	1	1	4	4	4
PRJNA396795	SRR5973333	17	17	2	1	1	1	4	4	4
PRJNA396796	SRR5973330	17	17	2	1	1	1	4	4	4
PRJNA396797	SRR5973347	17	17	2	1	1	1	4	4	4
PRJNA396798	SRR5973352	1824	1824	2	1	1	1	12	1	34
PRJNA396800	SRR5973320	1876	1876	2	5	1	1	12	1	18
PRJNA396801	SRR5973351	188	188	3	1	1	3	4	28	39
PRJNA396802	SRR5973266	1933	1933	2	1	1	1	3	1	9
PRJNA396803	SRR5973255	20	20	2	3	1	1	4	4	4
PRJNA396804	SRR5973331	20	20	2	3	1	1	4	4	4
PRJNA396805	SRR5973329	20	20	2	3	1	1	4	4	4
PRJNA396806	SRR5973387	20	20	2	3	1	1	4	4	4

PRJNA396807	SRR5973370	20	20	2	3	1	1	4	4	4
PRJNA396808	SRR5973410	200	200	2	1	2	1	12	1	68
PRJNA396809	SRR5973402	218	218	2	3	1	1	9	4	12
PRJNA396810	SRR5973338	2217	2217	2	1	2	3	26	4	25
PRJNA396811	SRR5973408	2267	2267	2	1	2	1	1	1	38
PRJNA396812	SRR5973385	2330	2330	4	1	1	1	3	7	6
PRJNA396813	SRR5973397	25	25	2	1	1	1	10	4	13
PRJNA396814	SRR5973248	258	258	3	3	1	1	1	1	79
PRJNA396815	SRR5973283	258	258	3	3	1	1	1	1	79
PRJNA396816	SRR5973279	258	258	3	3	1	1	1	1	79
PRJNA396817	SRR5973271	258	258	3	3	1	1	1	1	79
PRJNA396818	SRR5973336	258	258	3	3	1	1	1	1	79
PRJNA396819	SRR5973319	258	258	3	3	1	1	1	1	79
PRJNA396820	SRR5973317	258	258	3	3	1	1	1	1	79
PRJNA396821	SRR5973294	258	258	3	3	1	1	1	1	79
PRJNA396822	SRR5973291	258	258	3	3	1	1	1	1	79
PRJNA396823	SRR5973289	258	258	3	3	1	1	1	1	79
PRJNA396824	SRR5973287	258	258	3	3	1	1	1	1	79
PRJNA396825	SRR5973400	258	258	3	3	1	1	1	1	79
PRJNA396826	SRR5973382	258	258	3	3	1	1	1	1	79
PRJNA396827	SRR5973381	258	258	3	3	1	1	1	1	79
PRJNA396828	SRR5973357	261	261	2	1	1	1	4	27	12
PRJNA396830	SRR5973240	307	307	4	1	2	52	1	1	7
PRJNA396831	SRR5973247	307	307	4	1	2	52	1	1	7
PRJNA396832	SRR5973282	307	307	4	1	2	52	1	1	7
PRJNA396833	SRR5973280	307	307	4	1	2	52	1	1	7
PRJNA396834	SRR5973339	307	307	4	1	2	52	1	1	7
PRJNA396835	SRR5973322	307	307	4	1	2	52	1	1	7
PRJNA396836	SRR5973288	307	307	4	1	2	52	1	1	7
PRJNA396837	SRR5973396	307	307	4	1	2	52	1	1	7
PRJNA396838	SRR5973380	307	307	4	1	2	52	1	1	7
PRJNA396839	SRR5973379	307	307	4	1	2	52	1	1	7

PRJNA396840	SRR5973376	307	307	4	1	2	52	1	1	7
PRJNA396841	SRR5973373	307	307	4	1	2	52	1	1	7
PRJNA396842	SRR5973361	307	307	4	1	2	52	1	1	7
PRJNA396843	SRR5973355	307	307	4	1	2	52	1	1	7
PRJNA396844	SRR5973284	323	323	2	1	1	1	9	1	93
PRJNA396845	SRR5973332	35	35	2	1	2	1	10	1	19
PRJNA396846	SRR5973389	35	35	2	1	2	1	10	1	19
PRJNA396847	SRR5973368	35	35	2	1	2	1	10	1	19
PRJNA396848	SRR5973278	36	36	2	1	2	1	7	1	7
PRJNA396849	SRR5973309	36	36	2	1	2	1	7	1	7
PRJNA396850	SRR5973409	36	36	2	1	2	1	7	1	7
PRJNA396862	SRR5973241	393	393	4	1	1	1	9	5	9
PRJNA396863	SRR5973393	405	405	2	1	62	3	10	4	110
PRJNA396864	SRR5973311	412	412	2	1	2	1	9	1	112
PRJNA396865	SRR5973371	429	429	2	1	2	1	9	1	116
PRJNA396866	SRR5973263	433	433	2	1	2	60	7	1	7
PRJNA396867	SRR5973254	45	45	2	1	1	6	7	1	12
PRJNA396868	SRR5973276	45	45	2	1	1	6	7	1	12
PRJNA396870	SRR5973296	45	45	2	1	1	6	7	1	12
PRJNA396871	SRR5973292	45	45	2	1	1	6	7	1	12
PRJNA396872	SRR5973372	45	45	2	1	1	6	7	1	12
PRJNA396873	SRR5973327	466	466	2	1	2	1	10	50	120
PRJNA396874	SRR5973407	466	466	2	1	2	1	10	50	120
PRJNA396876	SRR5973239	492	492	4	7	1	1	9	4	17
PRJNA396877	SRR5973246	492	492	4	7	1	1	9	4	17
PRJNA396878	SRR5973275	492	492	4	7	1	1	9	4	17
PRJNA396879	SRR5973316	492	492	4	7	1	1	9	4	17
PRJNA396880	SRR5973301	502	502	2	53	3	1	10	4	18
PRJNA396881	SRR5973313	514	514	2	1	1	1	8	1	9
PRJNA396882	SRR5973392	528	528	32	5	1	1	9	4	18
PRJNA396883	SRR5973386	636	636	2	5	1	1	4	1	4
PRJNA396884	SRR5973404	65	65	2	1	2	1	10	4	13

PRJNA396885	SRR5973335	70	70	2	6	17	1	20	10	25
PRJNA396886	SRR5973314	753	753	14	1	2	1	135	4	12
PRJNA396887	SRR5973348	753	753	14	1	2	1	135	4	12
PRJNA396888	SRR5973257	774	774	2	1	1	17	4	16	4
PRJNA396889	SRR5973362	8	8	4	1	1	1	1	5	6

Table S10. The summary of WGS *K. pneumoniae* samples used for pipeline testing and results of testing

Colour	Meaning
(green)	non-homozygous sites detected
(orange)	predicted and expected ST do not match
(light yellow)	variants to not match completely with closest allele
